# The trajectory of head circumference and neurodevelopment in very preterm newborns during the first two years of life: a cohort study

**DOI:** 10.1016/j.jped.2024.04.005

**Published:** 2024-05-25

**Authors:** Maria Luciana de Siqueira Mayrink, Letícia Duarte Villela, Maria Dalva Barbosa Baker Méio, Fernanda Valente Mendes Soares, Andrea Dunshee de Abranches, Sylvia Reis Gonçalves Nehab, Ana Beatriz Rodrigues Reis, Leticia Baptista de Paula Barros, Maura Calixto Cecherelli de Rodrigues, Saint-Clair Gomes Junior, Maria Elisabeth Lopes Moreira

**Affiliations:** a Instituto Nacional de Saúde da Mulher, da Criança e do Adolescente Fernandes Figueira (IFF/FIOCRUZ), Rio de Janeiro, RJ, Brazil; b Pós-Graduação em Pesquisa Aplicada à Saúde da Criança e da Mulher, IFF/FIOCRUZ, Rio de Janeiro, RJ, Brazil; c Universidade do Estado do Rio de Janeiro (UERJ), Departamento de Pediatria da Faculdade de Ciências Médicas, Rio de Janeiro, RJ, Brazil

**Keywords:** Head circumference, Child development, Preterm infants, Follow-up

## Abstract

**Objective:**

To evaluate the growth trajectory of head circumference and neurodevelopment, and to correlate head circumference with cognitive, language, and motor outcomes during the first two years.

**Method:**

Prospective cohort study in a tertiary hospital including 95 newborns under 32 weeks or 1500 g. Neonates who developed major neonatal morbidities were excluded. The head circumference was measured at birth, at discharge, and at term-equivalent age, 1, 3, 5, 12, 18, and 24 months of corrected age, and the Bayley Scales (Bayley-III) were applied at 12, 18 and 24 months of corrected age to assess cognitive, language and, motor domains. Scores below 85 were classified as mild/moderate deficits and scores below 70 as severe deficits. The association between head circumference Z score and Bayley scores was assessed using Pearson's correlation. The study considered a significance level of 0.05.

**Results:**

There was a decrease of -0.18 in the head circumference Z score between birth and discharge and the catch-up occurred between discharge and 1 month (an increase of 0.81 in the Z score). There was a positive correlation between head circumference and Bayley scores at 18 months. There was also a positive correlation between head circumference at discharge and at 5 months with the three domains of the Bayley.

**Conclusion:**

Serial measurements of head circumference provide knowledge of the trajectory of growth, with early catch-up between discharge and 1 month, as well as its association with neurodevelopment. Head circumference is therefore a valuable clinical marker for neurodevelopment, especially in very preterm newborns.

## Introduction

Insufficient head circumference (HC) growth is a predictor of neurodevelopmental disabilities and serial HC measurements can identify children at risk of brain development and growth deficits.^1^, ^2^, ^3^, ^4^, ^5^ Longitudinal assessment of HC growth allows indirect monitoring of brain development, as studies show a correlation between HC and total brain volume assessed by magnetic resonance imaging.^1^^,^^5^ Very preterm newborns have lower HC at term corrected age than full-term newborns, as well as lower intelligence quotient scores, probably due to genetic, nutritional, and prematurity-related issues and secondary morbidities, among other causes.^6^^,^^7^

The survival rate of very preterm newborns has increased over the last three decades, from 53 % in the early 1990s to 73 % in 2016–2017.^8^ In this period, the prevalence of children who survived without significant neurodevelopmental impairments at 2 years of age, such as cerebral palsy, deafness, and blindness, increased from 42 % to 62 %.^8^ However, this group of newborns still has a prevalence of 35 to 50 % of cognitive and behavioral deficits and poor school performance, which is a worrying issue for health and education.^9^^,^^10^

The first thousand days of life comprise a sensitive period of brain development and growth, in which HC correlates with brain volume, and is considered a clinical marker and "proxy" for brain development and intelligence.^1^^,^^5^^,^^11^^,^^12^ Advances in neuroimaging and neuroscience have identified a vulnerability with regional volumetric reduction of the immature brain, with neurobehavioral consequences, especially in academic, social, and emotional performance of prematurely born children.^7^^,^^13^^,^^14^

Recent research has highlighted the correlation between HC at birth and brain growth during neonatal hospitalization and early childhood with cognitive, motor, attentional, and executive control skills.^1^^,^^2^^,^^5^ Raghuram and colleagues, in a cohort study with 1973 newborns under 29 weeks, observed a correlation between lower HC growth during the neonatal period and the first 2 years of life with neurodevelopmental impairments.^3^

However, the most sensitive period of brain growth, within the first 2 years of life, is still unknown.^1^^,^^5^ Studies correlating HC and development in very preterm newborns generally include children with major neonatal morbidities, and currently, the “dysmaturational” issues of the development of an immature brain are very much associated with the environmental exposure that the very preterm newborn experiences in the neonatal period.^7^

Thus, this study aimed to evaluate the growth trajectory of HC and neurodevelopment in very preterm or very low birthweight children without major neonatal morbidities, correlating this HC trajectory with cognitive, language, and motor development during the first two years of corrected age.

## Methods

### Study design, setting, and participants

This study is part of the “Coorte Pré-Crescer”, a cohort study of ‘healthy’ preterm infants at the Instituto Fernandes Figueira/FIOCRUZ, Rio de Janeiro, Brazil. It was approved by the Research Ethics Committee of this institute (CAAE 00754612.9.0000.5269) and the participants' guardians signed the Informed Consent Form before the study began. December 2016 was chosen as the end of the study period, because from that date an ongoing cohort was started, including very preterm or very low birthweight infants with and without neonatal morbidity.

This study included newborns with a gestational age of <32 weeks or a birthweight of <1500 g, admitted between 2012 and 2016 to the Institute's Neonatal Intensive Care Unit (NICU), without congenital malformation, genetic syndrome and congenital infections. Neonates who developed intracranial hemorrhage III and IV, severe neurological impairment, bronchopulmonary dysplasia (use of oxygen beyond 36 weeks of corrected age), neonatal sepsis (positive blood culture), necrotizing enterocolitis (stages II and III of Bell's classification),^15^ patent ductus arteriosus with surgical repair, perinatal hemolytic disease, use of exclusive parenteral nutrition for >7 days were excluded. These conditions were referred to as “major neonatal morbidities” for the study.

### Assessments and data collection

Head circumference was measured at birth, at the time of hospital discharge, and at the corrected ages of term, 1, 3, 5, 12, 18, and 24 months, using an inextensible tape measure at the largest occipitofrontal circumference. The HC Z score for age and sex was calculated using the Fenton neonatal growth chart (2013)^16^ as a reference up to 50 weeks of corrected age and, after this age, the WHO chart (2006).^17^ HC gain (HC delta) was calculated by the difference in measurement between two moments. Gestational age at birth is considered the first-trimester ultrasound scan or the date of the last menstrual period. The corrected ages during the study were calculated considering the corrected age of 40 weeks as the corrected term age. A gain in standard deviation greater than 0.67 in each period indicated a clinically significant catch-up in head circumference growth.^18^

The outcome was cognitive, language, and motor development, assessed with the Bayley Developmental Scales (Bayley-III), applied by a psychologist with experience in child development at the follow-up appointments at 12, 18, and 24 months of corrected age. This instrument is considered the gold standard for identifying developmental deviations in children and is widely used to monitor preterm-born children's development. The composite score has a mean of 100 (± 15), values below 85 (−1 SD of the mean) indicate mild/moderate developmental deficit, and values below 70 (−2 SD of the mean) indicate severe developmental deficit.^19^

Maternal, neonatal, and follow-up variables were collected from medical records and during clinical appointments: maternal age, maternal schooling, hypertension, diabetes, multiple gestations, gestational age, type of delivery, gender, APGAR scores, birth weight, length and HC and their respective Z scores, small for gestational age (SGA - birthweight less than the 10th percentile or weight Z score less than −1.28), parenteral nutrition, recovery of birthweight, use of oxygen therapy, length of hospital stay, corrected age at discharge, length of breastfeeding and presence of the father in daily care. The socioeconomic and family profile was evaluated by the information on maternal schooling and the presence of the father in daily care.

The recommended nutritional guidelines were followed during the neonatal hospitalization.^20^^,^^21^ In the follow-up, the nutritional practices encouraged breastfeeding and healthy eating.

### Statistical analysis

EPIINFO software was used for database storage and the statistical analyses were performed using SPSS software version 23. Descriptive analysis included the mean and standard deviation for continuous variables and proportions for categorical variables. Pearson's correlation was used to verify the association between HC at birth, at the time of hospital discharge, at the corrected ages of term, 1, 3, 5, 12 and 18 months, and the scores of the Bayley-III Scales at 12 and 18 months of corrected age. For all the analyses, the study considered a significance level of 0.05.

## Results

**Figure 1:**
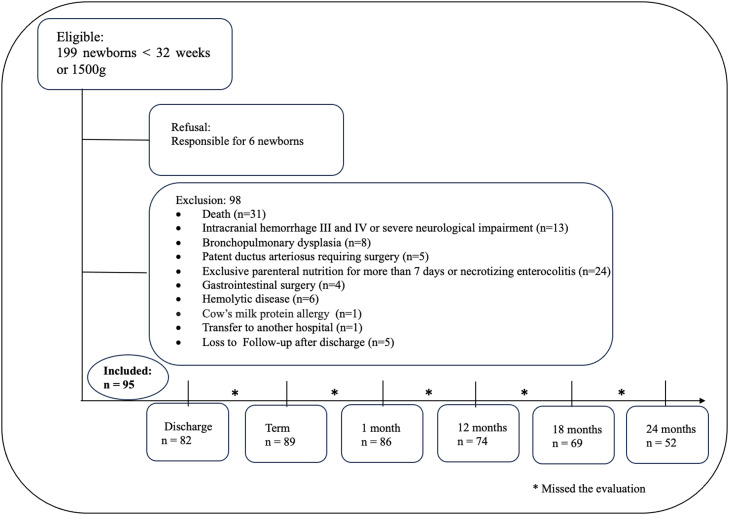
Flow diagram for study participants.

A total of 95 newborns without major neonatal morbidities were included in the study. Losses during follow-up occurred due to the missing Bayley-III appointments (Figure 1).

The mean gestational age was 30.1 weeks (± 2.2) and birthweight was 1275.8 g (± 312.4), with 27 % of newborns being SGA, and the average schooling of the mothers was 10.3 years (± 2.5) (Table 1).

**Table 1:** Maternal, neonatal and outpatient follow-up characteristics of the study population (*n* = 95).

Maternal characteristics	*n* (%)	Mean (SD)
Hypertension, *n* (%)	26 (30.2 %)	
Diabetes, *n* (%)	6 (7.0 %)	
Multiple gestations, *n* (%)	54 (57.4 %)	
Age (years), mean (SD)		27.4 (7.9)
Schooling (years of study), mean (SD)		10.3 (2.5)
**Neonatal and outpatient follow-up characteristics**	*n* (%**)**	**Mean (SD)**
Gestational age (weeks), mean (SD)		30.1 (2.2)
Cesarean section, *n* (%)	71 (76.3 %)	
Gender (male), *n* (%)	36 (38.3 %)	
APGAR 1, mean (SD)		7.1 (1.6)
APGAR 5, mean (SD)		8.6 (0.7)
Birthweight (g), mean (SD)		1275.8 (312.4)
Birth length (cm), mean (SD)		38.7 (3.0)
Birth head circumference (cm), mean (SD)		27.2 (2.3)
Birthweight Z score, mean (SD)		−0.56 (1.1)
Birth length Z score, mean (SD)		−0.37 (1.04)
Birth head circumference Z score, mean (SD)		−0.34 (0.92)
Small for gestational age, *n* (%)	26 (27.3 %)	
Parenteral nutrition (days), mean (SD)		10.6 (5.8)
Birthweight recovery (days of life), mean (SD)		14.5 (6.7)
Oxygen therapy (days), mean (SD)		12.1 (16.4)
Duration of neonatal hospitalization (days), mean (SD)		45.4 (19.1)
Corrected age at discharge (weeks), mean (SD)		36.3 (1.7)
Breastfeeding (months), mean (SD)		3.60 (3.64)
Father's presence in daily care, *n* (%)	65 (84.4 %)	

**Figure 2:**
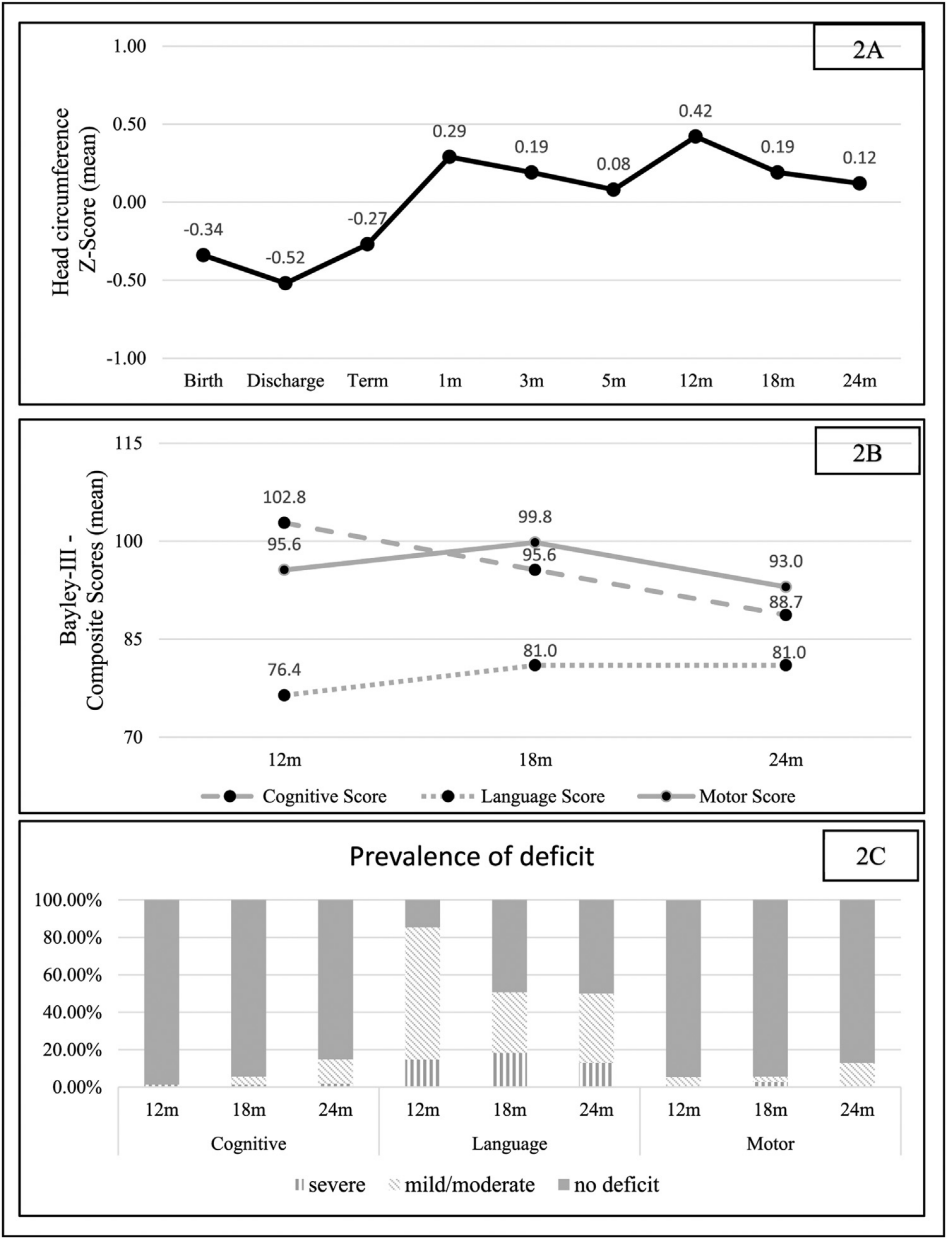
Head circumference and developmental trajectories (2A and 2B), and the prevalence (2C) of severe deficit (composite score below 70), mild/moderate deficit (composite score below 85), and adequate development (composite score greater than or equal to 85).

Regarding HC trajectory, there was a decrease in the Z score between birth and neonatal discharge, with a difference (delta) of - 0.18. The increase in the HC Z score was most evident between discharge and 1 month of corrected age, with a positive delta of 0.81 (catch-up). From this period until 2 years of corrected age, the HC Z score curve remained relatively stable (Figure 2).

The mean language scores remained below 85 at 12, 18, and 24 months of corrected age (1 SD below the mean), corresponding to a mild to moderate language deficit (Figure 2). However, the mean scores for the motor and cognitive domains remained within the expected range, although there was a downward trend in the mean cognitive scores towards the corrected age of two years (Figure 2).

The prevalence of total deficit (mild, moderate, or severe) found at 18 months of corrected age was 5.6 %, 50.7 %, and 5.6 % for the cognitive, language, and motor domains, respectively. At 24 months of corrected age, there was an increase in the prevalence of deficits in the cognitive and motor domains, to 14.9 % and 13.0 %, respectively, while for language the prevalence remained similar, at 50.0 %. It is noteworthy that in the cognitive domain, there is a progressive decrease in the proportion of children without deficits, but at the expense of a predominant increase in the proportion of moderate deficits; the same occurs in the language and motor domains (Figure 2).

There was a positive correlation between HC at hospital discharge and the assessment of cognitive, language, and motor domains performed at 12 months. The HC measurement at 5 months correlated positively with the three developmental domains assessed at 18 months of corrected age. Regarding cognitive and motor development, a positive correlation was found between HC at all the ages considered in this study and the Bayley-III assessment performed at 18 months of corrected age (Table 2).

**Table 2:** Correlation between head circumference Z score and Bayley-III Scales (Composite Scores) in very preterm or very low birth weight newborns at 12 and 18 months of corrected age.

			Domains of development					
			Cognitive		Language		Motor	
12 months		N	R	p-value	R	p-value	R	p-value
Head circumference	Birth	71	0,205	0,086	0,133	0,271	0,319**	0,007
	Discharge	67	0,260*	0,034	0,273*	0,025	0,287*	0,019
	Term	71	0,149	0,216	0,191	0,110	0,194	0,106
	1 month	72	0,146	0,220	0,142	0,236	0,195	0,101
	3 months	72	0,057	0,634	0,117	0,327	0,109	0,362
	5 months	61	−0,055	0,676	0,252*	0,050	0,066	0,615
	12 months	74	0,092	0,437	0,207	0,076	0,134	0,254

18 months		N	R	p-value	R	p-value	R	p-value
Head circumference	Birth	66	0,306*	0,013	0,133	0,286	0,311*	0,011
	Discharge	62	0,356**	0,005	0,195	0,130	0,330**	0,009
	Term	66	0,295*	0,016	−0,003	0,980	0,301*	0,014
	1 month	68	0,326**	0,007	0,103	0,403	0,315**	0,009
	3 months	67	0,290*	0,017	0,086	0,488	0,282*	0,021
	5 months	58	0,383**	0,003	0,307**	0,019	0,459**	0,000
	12 months	61	0,368**	0,004	0,230	0,075	0,304*	0,017
	18 months	69	0,265*	0,028	0,238*	0,049	0,314**	0,009

## Discussion

This study showed that preterm newborns experienced a decrease in their HC Z score between birth and hospital discharge when their catch-up began, and this accelerated HC growth continued up to 1 month of corrected age. This population evolved with a high prevalence of cognitive, language, and motor disabilities at 18 and 24 months of corrected age. There was a positive correlation between HC measurements at birth, hospital discharge, and at term, 1, 3, 5, 12, and 18 months of corrected age and development at 18 months of corrected age.

These results demonstrate the importance of serial assessment of HC as a clinical marker for developmental disorders. In this way, the longitudinal definition of growth restriction, considering HC measurements and not just weight, can contribute to the prediction of adverse neurodevelopmental outcomes throughout life.^22^

Neubauer et al. observed that very preterm newborns who evolved with "suboptimal" HC during the first two years of life had lower cognitive and psychomotor development scores at 12 and 24 months of corrected age compared with newborns who evolved with a normal HC.^4^ These authors showed a greater association between HC at 3 months and developmental delay at 12 and 24 months of corrected age and highlighted that HC at 3 months is a valuable marker of adverse neurodevelopmental outcomes.^4^ The current study found that HC at hospital discharge had a positive correlation with the cognitive, language, and motor domains at 12 months. There was also a positive correlation between HC at 5 months and the three developmental domains at 18 months.

This study showed that the HC gain between birth and 5 months and between birth and 24 months of corrected age was 14.6 cm and 20.5 cm, respectively. These findings are similar to those of Jaekel et al.’s study, in which they reported an increase of 14.5 cm and 20.2 cm in HC between birth and 5 months and between birth and 20 months of corrected age.^5^ These authors observed that both HC at birth and its growth between birth and 20 months and between 20 months and 4 years were predictive of intelligence at 6 years of age. This prediction is also influenced by the social and economic status of the family, which is linked to the development of brain regions related to language, memory, and cognition.^5^^,^^23^ Thus, there is significant growth in HC during the first two years of life, which is a critical window for brain development and growth.^5^^,^^24^^,^^25^^,^^26^

While the third trimester of intrauterine life is marked by accelerated brain growth,^11^ very preterm newborns miss this period inside the uterus. Additionally, they are exposed to adverse environmental factors and evolve with a decrease in HC growth until the period of hospital discharge. The current study found that the lowest HC Z score occurred at hospital discharge, with a slowdown in HC growth of −0.18 between birth and hospital discharge. Afterward there was a faster rate of HC growth, between hospital discharge and 1 month of corrected age (Z score delta of 0.81), characterizing a catch-up period. Neubauer et al. showed similar results in terms of the lowest HC Z score at discharge and the highest HC growth rate of 0.11 ± 1.2 between discharge and 3 months of corrected age^4^. This accelerated growth was lower than in the present study, which can be explained by the inclusion of newborns with neonatal morbidities and the improvement in the quality of neonatal care over the years. Sicard et al. also observed insufficient HC growth (negative Z-score delta of - 0.5) between birth and hospital discharge in newborns younger than 27 weeks but adequate HC growth in newborns older than 28–30 weeks.^2^

The study by Cho et al. with very preterm and SGA newborns showed a decrease in HC Z score between birth and 35 weeks, with developmental consequences at 18 months of corrected age and a significant HC catch-up between 35 and 40 weeks of corrected age that continued up to 4 months of corrected age.^27^ Cho et al. reported similar results to the present study since the neonates in the current study were discharged from the hospital at approximately 36 weeks of corrected age, at which point the HC catch-up started to become more noticeable up to 1 month of corrected age.

Insufficient HC growth during intrauterine and extrauterine life, and therefore lower HC at birth and at hospital discharge, respectively, can be considered predictors of unfavorable developmental outcomes, even in preterm newborns without major neonatal morbidities, as shown in this study at 12 and 18 months of corrected age. Corroborating this result, Sicard et al. demonstrated that the association of HC at birth lower than - 2DP and insufficient HC growth between birth and hospital discharge had a synergistic effect on the risk of developmental delay at 2 years.^2^ In their study, with 4046 newborns younger than 34 weeks, there was a negative correlation between the HC Z score at birth and HC growth until hospital discharge with neurodevelopmental delay at 2 years of corrected age.^2^ Selvanathan et al. observed similar findings of a correlation of HC at birth, with lower cognitive scores at 18 and 36 months of age, and worse outcomes in the intelligence and motor assessments at 4 years of age, regardless of postnatal diseases and the volume of diffuse white matter alterations.^1^

In addition, insufficient HC growth that persists after discharge from the NICU also contributes to negative neurodevelopmental outcomes.^1^^,^^3^^,^^27^ Raghuram et al. showed that babies who had insufficient HC growth (HC Z score delta < −2DP) between birth and 16–36 months of age had a higher risk of significant cognitive, language, and motor delay (Bayley-III score < 70) at this age, thus highlighting the importance of HC catch-up growth after hospital discharge and during the first months of life.^3^

Long-term follow-up studies with babies born very prematurely are therefore necessary, especially as neurodevelopmental impairments become more apparent as the children acquire higher-order skills.^28^ Consistent with the findings of the publication by Garfinkle et al., the current study demonstrated a trend towards greater detection of developmental delay as the age of 12 to 24 months advanced. Garfinkle et al. showed that assessment at a later age may be more accurate in diagnosing developmental deficits as tasks become more complex.^28^

The findings of this study showed that the prevalence of delay in the cognitive, language, and motor domains at 2 years of corrected age was 14.9 %, 50.0 %, and 13.0 %, respectively. The study by Valentini et al. also found a high prevalence of 30 % of cognitive delay at 4 and 24 months; 50 % of language delay at 4 and 24 months and 50 % of motor delay at 8 and 12 months.^29^ In Pierrat et al.'s study, the proportion of children born at 24–26 and 27–31 weeks' gestation who had at least one of the neurodevelopmental domains below the threshold at 2 years was 50.2 % and 40.7 %, respectively.^30^

In the last 2–3 decades, there has been a decrease in the incidence of severe neurodevelopmental disorders such as severe cerebral palsy, as well as the presence of severe neonatal morbidities such as cystic periventricular leukomalacia.^7^^,^^8^^,^^11^^,^^30^ This transition in the diagnosis of brain lesions, from major lesions such as extensive intracranial hemorrhages and cystic periventricular leukomalacia to the recognition of diffuse and sometimes microstructural changes in brain maturation, which can be subtle and underdiagnosed, may explain the increase in the survival of very preterm babies with a high and sustained incidence of cognitive impairment and behavioral and motor disorders with social and emotional repercussions throughout life.^7^^,^^11^^,^^14^^,^^29^^,^^30^ Diffuse and microstructural alterations are associated with reductions in the functional connectivity of frontoparietal and executive control neural networks, which predispose children born very preterm to deficits in intelligence, executive function, attention, processing speed, language skills, academic performance, and motor skills at school age.^9^^,^^14^

The strength of this study results from the longitudinal monitoring of HC and neurodevelopment in children born very preterm up to 2 years of age by an interprofessional team. It should be noted that serial assessment of HC is a rapid and low-cost technique that can easily be implemented in the clinical routine, from birth and during neonatal hospitalization, up to the first years of life. In addition, the HC measurement correlates with brain volume measured by nuclear resonance and allows early identification of risk and timely intervention to optimize neurodevelopment.

The study had some limitations, such as a loss of about 50 % at 24 months and the lack of a sample size calculation, although the use of an established sample allows access to longitudinal data and the ability to look at trends over time. Other limitations were the non-inclusion of newborns with significant morbidities and, thus, individual clinical challenges affecting both growth and neurodevelopmental trajectories. Therefore, the results cannot be generalized to the group of very preterm infants, as no critically ill infants were included. However, it was possible to observe the prevalence of neurodevelopmental deficits and the change in Bayley-III scores according to the age of the assessment. Another issue is that the study was carried out in a single center, and replication of the results in different contexts will allow greater applicability of head circumference assessment as an indicator of neurodevelopment. More multicenter follow-up studies into school age and adolescents should be encouraged to better understand the lifelong effects of prematurity in terms of socialization, behavior and learning.

In conclusion, the study highlights the importance of longitudinal growth assessment of HC from birth, during the neonatal period and the first months of life, especially in very preterm newborns who evolve with a high prevalence of developmental deficits. The catch-up of HC growth occurred very precociously, between discharge and 1 month of corrected age, and HC correlates with development at 12 and 18 months of corrected age. HC growth is a clinical predictor of neurodevelopment during childhood and growth-enhancing practices should be provided, such as promoting nutrition and encouraging the maintenance of breastfeeding, and guidelines that support the baby's stimulation, affection, and bonding with their caregivers during the hospitalization, the catch-up period and the valuable "first 1000 days of life".

## Conflicts of interest

The authors declare no conflicts of interest.

## References

[1] Selvanathan T., Guo T., Kwan E., Chau V., Brant R., Synnes A.R. (2022). Head circumference, total cerebral volume and neurodevelopment in preterm neonates. Arch Dis Child Fetal Neonatal Ed.

[2] Sicard M., Nusinovici S., Hanf M., Muller J.B., Guellec I., Ancel P.Y. (2017). Fetal and postnatal head circumference growth: synergetic factors for neurodevelopmental outcome at 2 years of age for preterm infants. Neonatology.

[3] Raghuram K., Yang J., Church P.T., Cieslak Z., Synnes A., Mukerji A. (2017). Head growth trajectory and neurodevelopmental outcomes in preterm neonates. Pediatrics.

[4] Neubauer V., Griesmaier E., Pehböck-Walser N., Pupp-Peglow U., Kiechl-Kohlendorfer U. (2013). Poor postnatal head growth in very preterm infants is associated with impaired neurodevelopment outcome. Acta Paediatr.

[5] Jaekel J., Sorg C., Baeuml J., Bartmann P., Wolke D. (2019). Head growth and intelligence from birth to adulthood in very preterm and term born individuals. J Int Neuropsychol Soc.

[6] Johnson M.J., Wootton S.A., Leaf A.A., Jackson A.A. (2012). Preterm birth and body composition at term equivalent age: a systematic review and meta-analysis. Pediatrics.

[7] Inder T.E., Volpe J.J., Anderson P.J. (2023). Defining the neurologic consequences of preterm birth. N Engl J Med.

[8] Cheong J.L.Y., Olsen J.E., Lee K.J., Spittle A.J., Opie G.F., Clark M. (2021). Temporal trends in neurodevelopmental outcomes to 2 years after extremely preterm birth. JAMA Pediatr.

[9] He L., Parikh N.A. (2015). Aberrant executive and frontoparietal functional connectivity in very preterm infants with diffuse white matter abnormalities. Pediatr Neurol.

[10] Luu T.M., Rehman Mian M.O., Nuyt A.M (2017). Long-term impact of preterm birth: neurodevelopmental and physical health outcomes. Clin Perinatol.

[11] Cormack B.E., Harding J.E., Miller S.P., Bloomfield F.H. (2019). The influence of early nutrition on brain growth and neurodevelopment in extremely preterm babies: a narrative review. Nutrients.

[12] Dupont C., Castellanos-Ryan N., Séguin J.R., Muckle G., Simard M.N., Shapiro G.D. (2018). The predictive value of head circumference growth during the first year of life on early child traits. Sci Rep.

[13] Zhang Y., Inder T.E., Neil J.J., Dierker D.L., Alexopoulos D., Anderson P.J. (2015). Cortical structural abnormalities in very preterm children at 7 years of age. Neuroimage.

[14] Anderson P.J., Treyvaud K., Neil J.J., Cheong J.L.Y., Hunt R.W., Thompson D.K. (2017). Associations of newborn brain magnetic resonance imaging with long-term neurodevelopmental impairments in very preterm children. J Pediatr.

[15] Bell M.J., Ternberg J.L., Feigin R.D., Keating J.P., Marshall R., Barton L. (1978). Neonatal necrotizing enterocolitis. Therapeutic decisions based upon clinical staging. Ann Surg.

[16] Fenton T.R., Kim J.H. (2013). A systematic review and meta-analysis to revise the Fenton growth chart for preterm infants. BMC Pediatr.

[17] WHO Multicentre Growth Reference Study Group (2006). WHO Child Growth Standards based on length/height, weight and age. Acta Paediatr Suppl.

[18] Ong K.K., Ahmed M.L., Emmett P.M., Preece M.A., Dunger D.B. (2000). Association between postnatal catch-up growth and obesity in childhood: prospective cohort study. BMJ.

[19] Bayley N. (2017).

[20] Senterre T., Koletzko B, Poindexter B, Uauy R (2014). Nutritional Care of Preterm Infants: Scientific Basis and Practical Guidelines.

[21] Embleton N.D., Simmer K., Koletzko B, Poindexter B, Uauy R (2014). Nutritional Care of Preterm Infants: Scientific Basis and Practical Guidelines.

[22] Fenton T.R., Cormack B., Goldberg D., Nasser R., Alshaikh B., Eliasziw M. (2020). Extrauterine growth restriction" and "postnatal growth failure" are misnomers for preterm infants. J Perinatol.

[23] Benavente-Fernández I., Siddiqi A., Miller S.P. (2020). Socioeconomic status and brain injury in children born preterm: modifying neurodevelopmental outcome. Pediatr Res.

[24] Cunha A.J., Leite Á.J., Almeida I.S. (2015). The pediatrician's role in the first thousand days of the child: the pursuit of healthy nutrition and development. J Pediatr.

[25] Harvard University (2007). Brief: the Science of Early Childhood Development [Internet].

[26] Bhutta Z.A., Guerrant R.L., Nelson C.A. (2017). Neurodevelopment, nutrition, and inflammation: the evolving global child health landscape. Pediatrics.

[27] Cho H., Kim E.K., Song I.G., Heo J.S., Shin S.H., Kim H.S. (2021). Head growth during neonatal intensive care unit stay is related to the neurodevelopmental outcomes of preterm small for gestational age infants. Pediatr Neonatol.

[28] Garfinkle J., Khairy M., Simard M.N., Wong J., Shah P.S., Luu T.M. (2024). Corrected age at bayley assessment and developmental delay in extreme preterms. Pediatrics.

[29] Valentini N.C., de Borba L.S., Panceri C., Smith B.A., Procianoy R.S., Silveira R.C. (2021). Early detection of cognitive, language, and motor delays for low-income preterm infants: a Brazilian cohort longitudinal study on infant neurodevelopment and maternal practice. Front Psychol.

[30] Pierrat V., Marchand-Martin L., Arnaud C., Kaminski M., Resche-Rigon M., Lebeaux C. (2017). Neurodevelopmental outcome at 2 years for preterm children born at 22 to 34 weeks' gestation in France in 2011: EPIPAGE-2 cohort study. BMJ.

